# Evaluating effect of metallic ions on aggregation behavior of β-amyloid peptides by atomic force microscope and surface-enhanced Raman Scattering

**DOI:** 10.1186/s12938-021-00972-7

**Published:** 2021-12-30

**Authors:** Yang Xie, Lin Yu, Yuna Fu, Heng Sun, Jianhua Wang

**Affiliations:** 1 Pharmaceutical Engineering Center, Chongqing Medical and Pharmaceutical College, Chongqing, 401331 China; 2 Key Laboratory of Biorheological Science and Technology, Ministry of Education, and Institute of Biochemistry and Biophysics, College of Bioengineering, Chongqing University, Chongqing, 400044 China

**Keywords:** β-Amyloid peptides, Self-assembly monolayers, Atomic force microscopy, Surface-enhanced Raman Scattering

## Abstract

**Background:**

Excessive aggregation of β-amyloid peptides (Aβ) is regarded as the hallmark of Alzheimer’s disease. Exploring the underlying mechanism regulating Aβ aggregation remains challenging and investigating aggregation events of Aβ in the presence and absence of metallic ions at molecular level would be meaningful in elucidating the role of metal cations on interactions between Aβ molecules. In this study, chemical self-assembled monolayer (SAM) method was employed to fabricate monolayer of β-amyloid peptides Aβ42 on gold substrate with a bolaamphiphile named 16-Mercaptohexadecanoic acid (MHA). Firstly, the samples of gold substrate (blank control), the MHA-modified substrate, and the Aβ42-modified substrate were detected by X-ray photoelectron spectroscopy (XPS) to track the self-assembly process. Aggregation behaviors of Aβ42 before and after metallic ions (Zn^2+^, Ca^2+^, Al^3+^) treatment were monitored by atomic force microscopy (AFM) and the interaction between Aβ42 and metallic ions (Zn^2+^, Ca^2+^, Al^3+^) was investigated by surface-enhanced Raman Scattering (SERS).

**Results:**

The XPS spectra of binding energy of gold substrate (blank control), the MHA-modified substrate, and the Aβ42-modified substrate are well fitted with the corresponding monolayer’s composition, which indicates that Aβ42 monolayer is well formed. The recorded surface morphology of different experimental groups obtained by AFM showed markedly different nanostructures, indicating occurrence of aggregation events between Aβ42 molecules after adding metal ions to the solution. Compared to the control group, the presence of metallic ions resulted in the increased size of surface structures on the observed 3D topography. Besides, the intermolecular rupture force of Aβ42 increased with the addition of metallic ions. Further study by SERS showed that the Raman strength of Aβ42 changes significantly after the metal cation treatment. A considerable part of the amide bonds interacts with metal cations, leading to a structural change, which is characterized by the weakened β-fold Raman peak.

**Conclusion:**

The AFM imaging results suggest that aggregation events occurred between Aβ42 molecules with the addition of metal cations. In addition, the results of force tests indicate that the presence of metallic ions could promote adhesion between Aβ42 molecules, which is likely to be the trigger for aggregation behavior of Aβ42. Furthermore, the effect of metallic cations on the conformational change of Aβ42 studied by SERS supported the results obtained by AFM. Taken together, the results showed that the presence of substoichiometric metal cations promotes aggregation behavior between Aβ42 molecules on the substrate at pH 7.4.

## Background

In recent years, Alzheimer’s disease (AD) is paid more and more attention for its terrible influence on the health of the elderly. Amyloid plaques with high density of β-amyloid peptides (Aβ) in the brain tissue of patients have been reported to be the major pathological feature of AD [1]. It was reported that the concentration of deposited Aβ in the cerebral cortex correlates with the degree of dementia and synaptic loss [2]. Main components of the plaques were studied to be Aβ40 and Aβ42, which are two homologous isomers of Aβ [3]. While Aβ40 is the most abundant homologous isomers ever discovered, Aβ42 has been reported to be the most toxic [4, 5]. Hence, the article focuses on the aggregation of Aβ42, whose neurotoxicity has essential correlation with the pathology of AD [6]. There remains a key question in the pathology of AD: what are the risk factors that affect the aggregation of Aβ? In recent years, several neurotoxic metal ions were proposed as affecting factors in the misfolding and aggregation processes of Aβ [7]. It has been found through the autopsy of AD patients that abnormally high concentrations of Zn^2+^ and Ca^2+^ are present along with Aβ in the senile plaques of AD [8, 9], where Al^3+^ is also detected [10].

Exploring the underlying mechanism regulating Aβ aggregation remains challenging and the mechanism of metal cations’ effect on Aβ remains elusive. Until now, the lack of study on revealing molecular events of Aβ raised a controversy about whether metal cations are helpful to the aggregation of Aβ molecules. Therefore, a study on aggregation events of Aβ in the presence and absence of metal ions at molecular level would be of great significance in terms of pathology and methodology. In recent years, atomic force microscopy (AFM) has been widely used for studying the interaction between molecules, especially protein–protein interaction [11, 12]. Since the turn of the century, new perspectives have been opened with the advent of AFM in the investigation of biomolecular interactions [13]. Up to now, some research methods have been developed to study protein–protein interactions, such as surface plasmon resonance (SPR) [14], enzyme immunoassay (EIA) [15], surface force apparatus (SFA) [16], and atomic force microscopy (AFM) [17]. Compared with other methods, AFM has the advantage of obtaining images with high spatial resolution and carrying out measurements under near-physiological conditions [18]. Consequently, AFM opens novel avenues for studying pathways of interactions between proteins and makes the study of physical behaviors of proteins achievable. In addition, along with the advantage of obtaining protein molecules’ topographies on nanoscale, AFM enables researchers to monitor the aggregation behaviors between Aβ monomers. This study focuses on the effects of several metal ions (Zn^2+^, Ca^2+^, Al^3+^) on Aβ42 aggregation. The three-dimensional morphology measured by AFM has high spatial resolution. All experiments were carried out under near-physiological conditions and a low ion concentration, closer to physiologically relevant values, is applied. By using AFM, it is achievable to concentrate on what we can find by “looking” at the protein molecules and analyze biological phenomena [19].

**Fig. 1:**
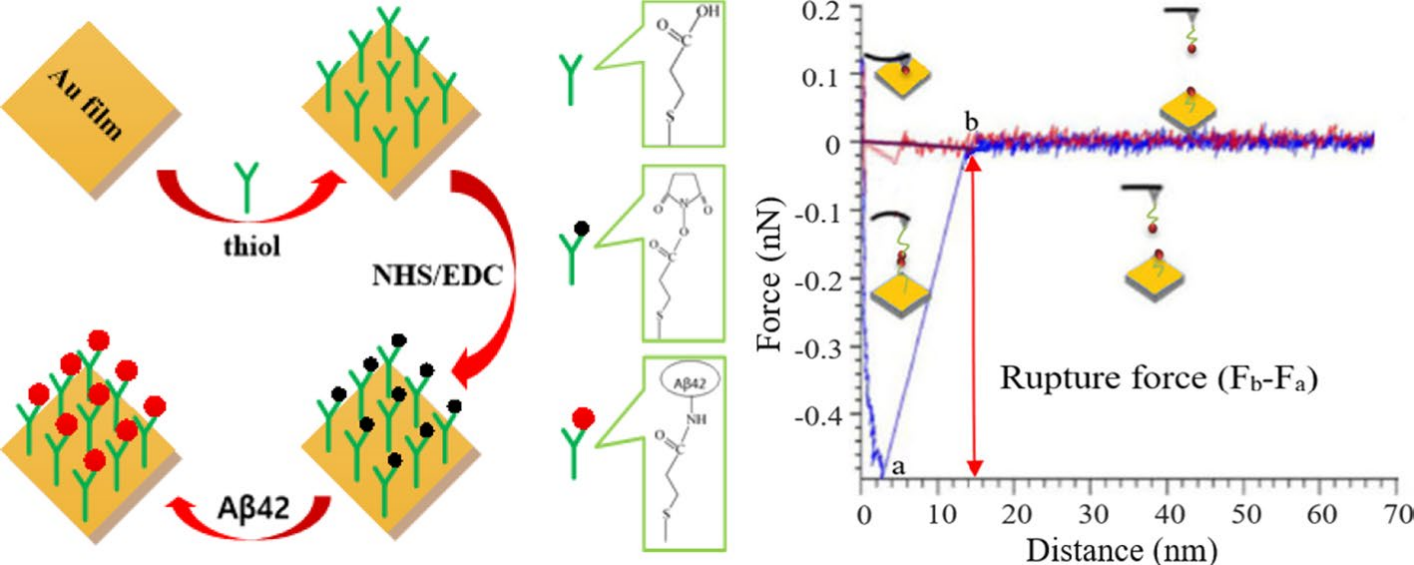
Experimental setup of AFM. **a** A schematic diagram of mercaptan self-assembly method, which shows covalent attachment of Aβ42 on the carboxyl terminal of thiol-modified gold substrate. **b** A typical force–distance curve with recorded rupture force (*F*_*a*_ − *F*_*b*_). Aβ42 is immobilized on the Au-modified mica surface via MHA linker. The counterpart Aβ42 is immobilized on the MHA functionalized Au-coated tip

However, the main challenge with AFM testing is the sample preparation, especially the stabilization of protein molecules onto the gold substrate. In this study, the possibility of investigating molecular events in physiological solution is realized through a sample preparation method called self-assembly monolayer (SAM), which has been developed over the last two decades and widely applied by biologists and chemists [20, 21]. To achieve SAM chemically, a cleaned gold substrate should be immersed in a solution of thiols (16-Mercaptohexadecanoic acid (MHA)), which is followed by spontaneous reaction between the thiol and gold. Gradually, MHA monolayer on the gold surface with ordered and stable bonds (Au–S bond) is formed. Afterward, the carboxyl terminus of MHA is activated by 1-ethyl-3-(dimethylaminopropyl)carbodiimide hydrochloride (EDC) and *N*-Hydroxysulfosuccinimide (NHS) and then immersed into protein (Aβ42) solution. Eventually, a stable and ordered monolayer of Aβ42 on the thiol-modified gold surface is formed. The mechanism of mercaptan self-assembly method is illustrated in Fig. 1a.

In order to track the formation of Aβ monolayer, X-ray photoelectron spectroscopy (XPS) was employed. The composition characteristics of the gold surface, the Aβ42 monolayer, and the MHA film were studied. Next, we monitored the nanostructure changes of Aβ42 in the presence and absence of metallic ions (Zn^2+^, Ca^2+^, Al^3+^) by employing AFM imaging and performed force measurement after Aβ42 aggregation. What worth mentioning is that Aβ42 molecules must be immobilized on the surface of the gold-coated probe tip and the substrate before force test. In this study, the interaction of Aβ42 monolayers is characterized by rupture force (Fig. 1b) between the probe tip and substrate [22]. Furthermore, the interaction between metallic ions and Aβ is probed and discussed by Surface-Enhanced Raman Scattering (SERS), which is an emerging sample surface analysis technique [23]. The changes of chemical bonds and groups in molecules lead to different molecular rotation or vibration states, which can be judged by the change of Raman scattering light frequency [24]. For proteins, Raman scattering can obtain not only important information about amino acid composition but also secondary structure information, such as β-sheet and α-helix. It has been reported that the Raman cross sections of Au are enhanced to some extent when they adsorb different molecules [25]. Because this effect occurs in the metal adsorbed molecular system, many important processes, such as surface studies, are related to it. The purpose of SERS study is to quantitatively characterize the effect of metal ions on the conformational transition of Aβ42, and to probe the role of these metal ions in the process of abnormal aggregation of Aβ42. Combined with the results of AFM study, the aggregation behavior of Aβ in the absence and presence of metallic ions was elucidated.

## Results

### Characterization of bare Gold, MHA Film, and Aβ42 monolayer by XPS

**Fig. 2:**
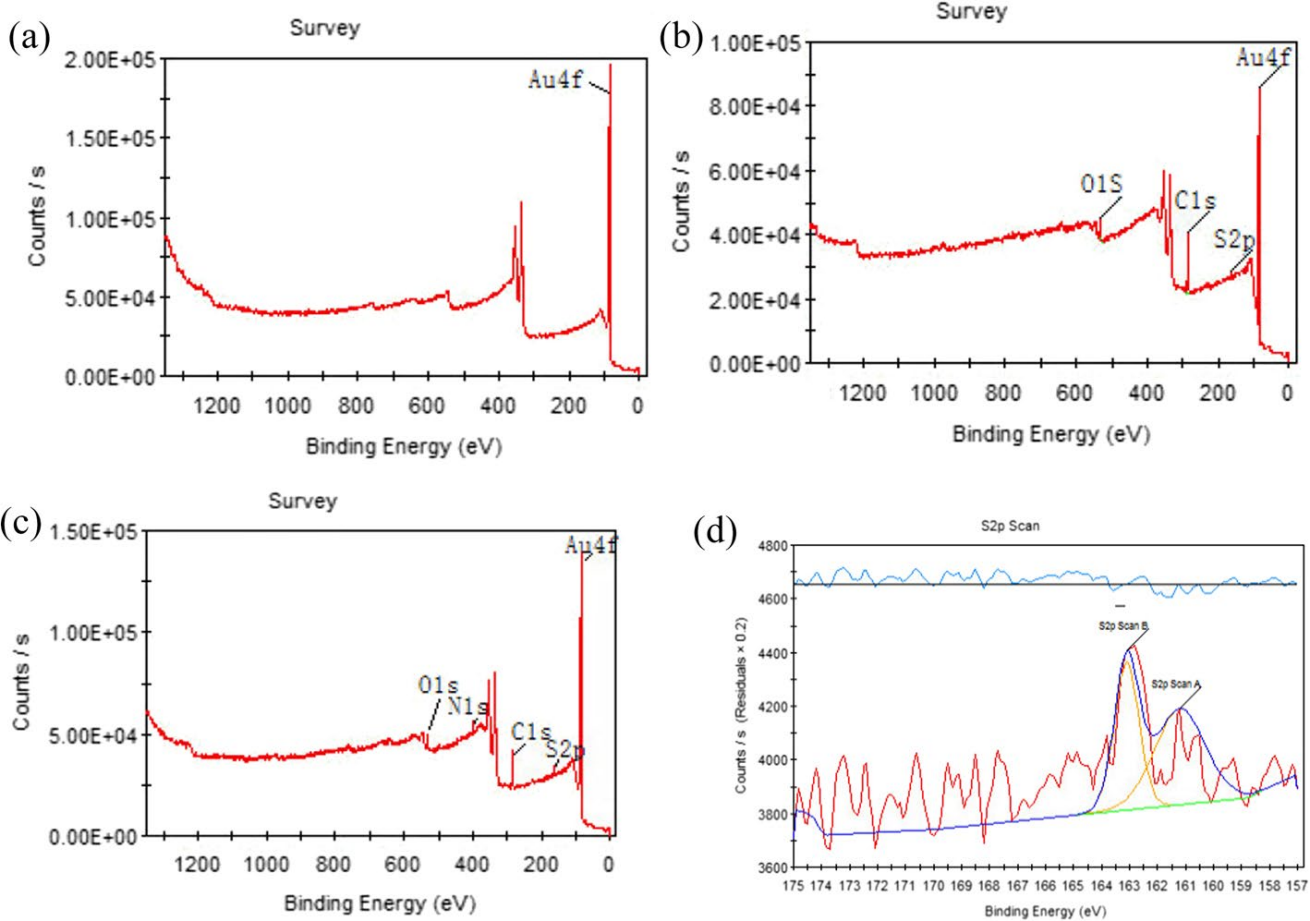
**a** Binding energy spectra of bare gold; **b** Binding energy spectra of MHA; **c** Binding energy spectra of Aβ42 monolayer; **d** Binding energy spectra of S2p after MHA modification. The spectra were measured at 25 °C

The surfaces of different samples (blank substrate, MHA films, Aβ42 monolayer) were investigated by XPS and all measurements should be repeated 3 times for each sample. Full spectrum of elements obtained by XPS showed changed element binding energy of the bare gold, MHA-modified, and the Aβ42-modified gold sample. Figure 2 shows the full XPS spectra of the three surfaces, namely, binding energy spectra of bare gold (a), binding energy spectra of MHA (b), and binding energy spectra of Aβ42 monolayer (c). Besides, binding energy spectra of S2p after MHA modification (d) were recorded.

### AFM imaging and force measurement

**Fig. 3:**
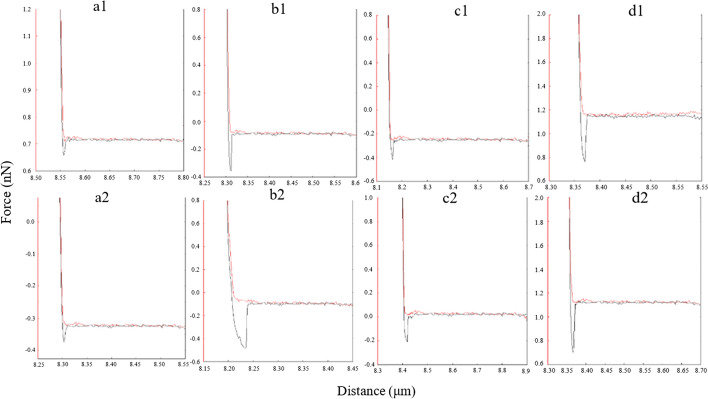
Representative force–distance curves measured in different experimental groups **a1** Aβ42 monolayer incubated in blank solution for 24 h; **a2** Aβ42 monolayer incubated in blank solution for 48 h; **b1** Aβ42 monolayer incubated in the presence of Zn^2+^ for 24 h; **b2** Aβ42 monolayer incubated in the presence of Zn^2+^ for 48 h; **c1** Aβ42 monolayer incubated in the presence of Ca^2+^ for 24 h; **c2** Aβ42 monolayer incubated in the presence of Ca^2+^ for 48 h; **d1** Aβ42 monolayer incubated in the presence of Al^3+^ for 24 h; **d2** Aβ42 monolayer incubated in the presence of Al^3+^ for 48 h. (red line––approach trace, blue line––retract trace)

**Fig. 4:**
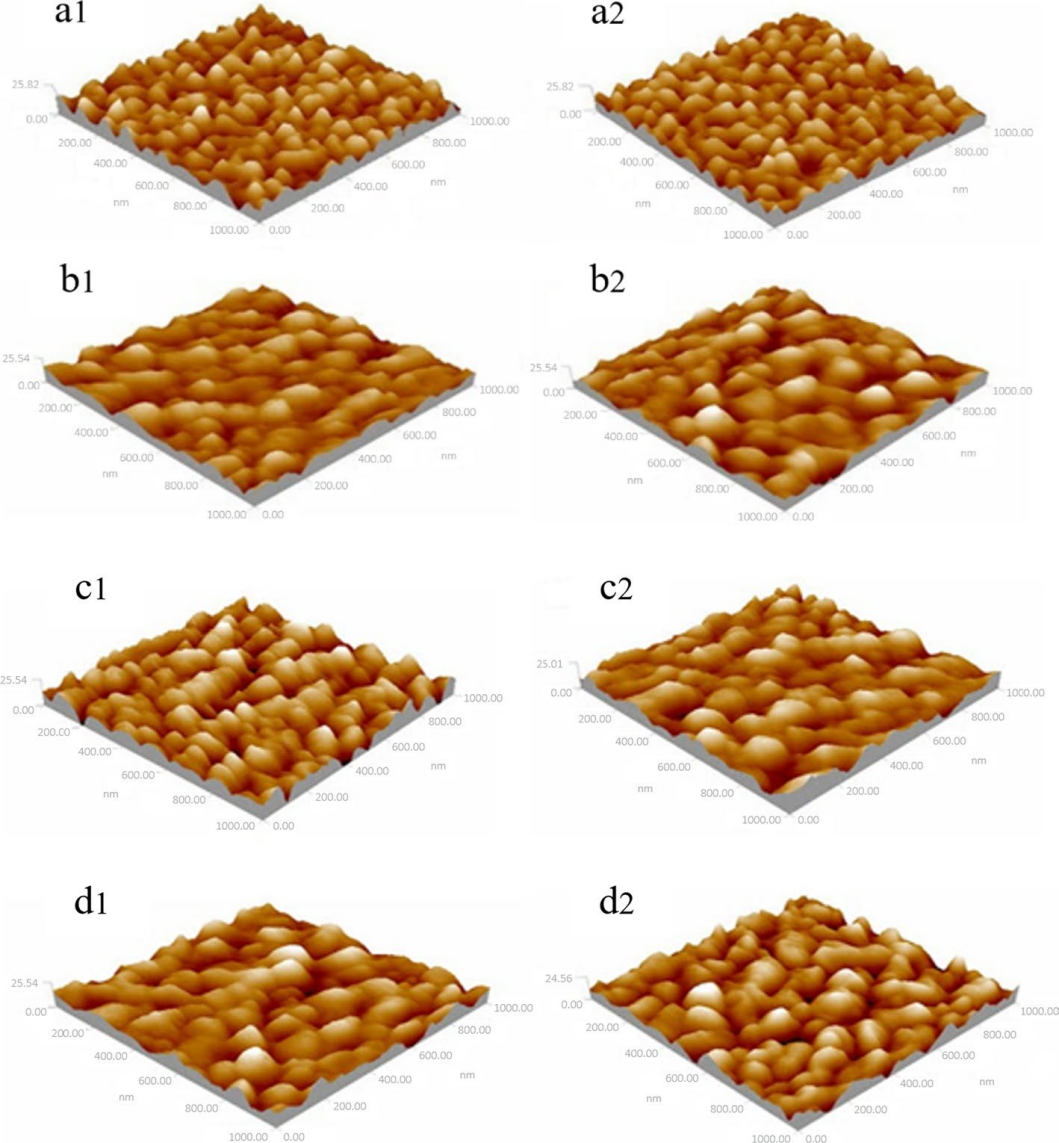
**a1** Topography of Aβ42 monolayer imaged by AFM in PBS at the incubation time of 24 h; **a2** Topography of Aβ42 monolayer imaged by AFM in PBS at the incubation time of 48 h; **b1** Topography of Aβ42 monolayer in the presence of Zn^2+^ at the incubation time of 24 h; **b2** Topography of Aβ42 monolayer in the presence of Zn^2+^ at the incubation time of 48 h; **c1** Topography of Aβ42 monolayer in the presence of Ca^2+^ at the incubation time of 24 h; **c2** Topography of Aβ42 monolayer in the presence of Ca^2+^ at the incubation time of 48 h; **d1** Topography of Aβ42 monolayer in the presence of Al^3+^ at the incubation time of 24 h; **d2** Topography of Aβ42 monolayer in the presence of Al^3+^ at the incubation time of 48 h. The concentration of metal ions is 10 μM (the scanning range is 1000 nm × 1000 nm) (pH 7.4)

Figure 3 shows the histogram of average diameter of the nanostructures on the images of the Aβ42 monolayer in blank solution, 10 µM Zn^2+^ solution, 10 µM Ca^2+^ solution, and 10 µM Al^3+^ solutions, respectively. Firstly, a three-dimensional topography of Aβ42-modified substrate was recorded in PBS by AFM as the control experiment (Fig. 4(a1)). The topography image of Aβ42 monolayer in the absence of metallic cations displayed homogeneous sharp granular-like structure after incubating for 24 h or 48 h (Fig. 4(a1)). However, the topography of Aβ42 monolayer in the presence of Zn^2+^, Ca^2+^, and Al^3+^ showed irregular smooth spheroid-like structures after 24 h (Fig. 4(b1), (c1), (d1)). With the increase of incubation time to 48 h, globular features were still observed on the surface of Aβ42 monolayer treated with metallic cations (Fig. 4(b2), (c2), (d2)).

**Fig. 5:**
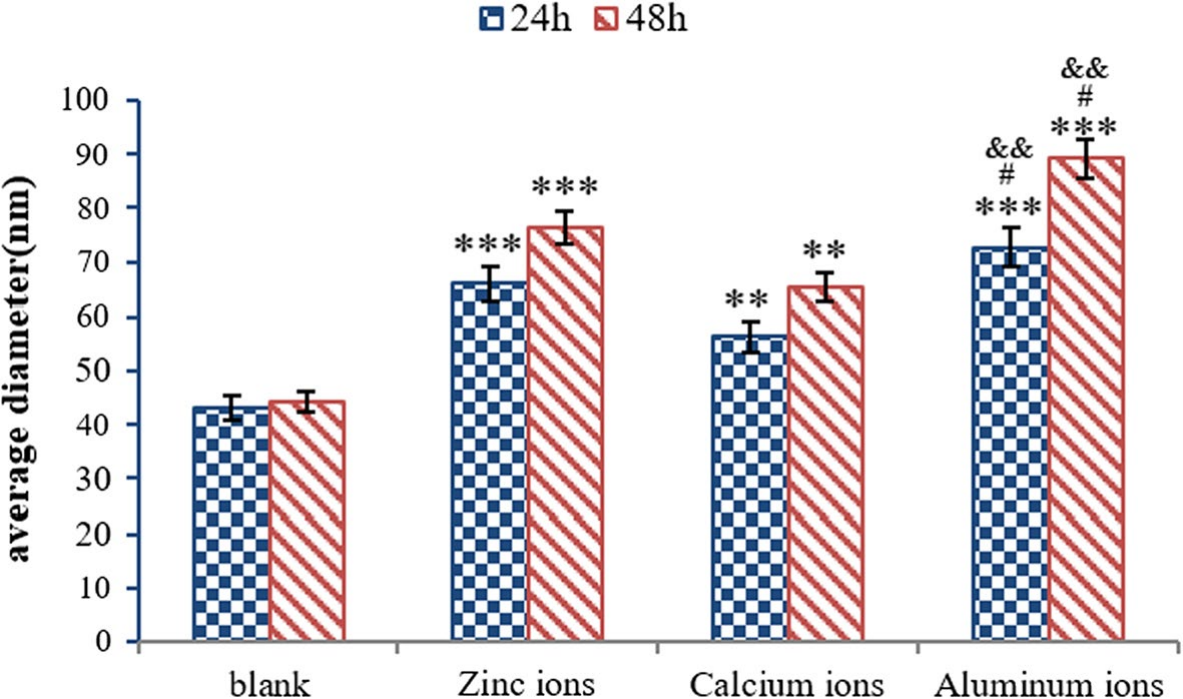
Average particle size of Aβ42 monolayer in the absence and presence of metallic ions (Zn^2+^, Ca^2+^ and Al^3+^) at different incubation time points. All experiments were performed ten times (n = 10). ***P < 0.001, **P < 0.005 vs. control; ^#^*P* < 0.01 vs. PBS + Zn^2+^; ^&&^*P* < 0.005 vs. PBS + Ca^2+^

The sizes of the nanostructures on the surface of each sample were characterized by average diameter by using software analysis (as shown in Fig. 5). The average size of the observed nanostructure in control group was calculated to be 43.12 nm at the incubation time of 24 h (Fig. 5, blue bar) and 44.32 nm at the incubation time of 48 h (Fig. 5, red bar), respectively. After treated with Zn^2+^, the average size of the surface nanostructures increased to 65.87 nm at the incubation time of 24 h and 76.13 nm at the incubation time of 48 h, respectively. For Aβ42 monolayer treated with Ca^2+^, the average size of the surface nanostructures was calculated to be 56.31 nm at the incubation time of 24 h and 65.34 nm at the incubation time of 48 h, respectively. It is worth noting that in the presence of Al^3+^, the average size of nanostructures observed on the image significantly increased to 72.67 nm at the incubation time of 24 h and 89.12 nm at the incubation time of 48 h.

In addition, the surface roughness of each topography is characterized by an average roughness (*R*_n_) (shown in Table 1). By applying software analysis, it was found that the surface roughness of Aβ42 monolayer in different experimental groups varied significantly. The change of surface particles can be characterized by average roughness *R*_n_. The results showed that the average roughness of control group (in physiological solution) was 1.958 at the incubation time of 24 h and little change of *R*_n_ was observed at the incubation time of 48 h. For Aβ42 monolayer incubated in 10 µM Zn^2+^, Ca^2+^ and Al^3+^ solutions for 24 h, *R*_n_ was calculated to be 1.762, 1.82, and 1.672, respectively. For Aβ42 monolayer incubated in 10 µM in 10 µM Zn^2+^, Ca^2+^, and Al^3+^ solutions for 48 h, the *R*_n_ was calculated to be 1.54, 1.622, and 1.412, respectively.

**Table 1:** Average roughness (*R*_*n*_) of Aβ42 monolayer in the absence and presence of metallic ions at different incubation time points

sample	*R*_*n*_	sample	*R*_*n*_
Control(24 h)	1.958	Control(48 h)	1.954
Zn^2+^(24 h)	1.762	Zn^2+^(48 h)	1.54
Ca^2+^ (24 h)	1.82	Ca^2+^ (48 h)	1.622
Al^3+^(24 h)	1.672	Al^3+^(48 h)	1.412

**Fig. 6:**
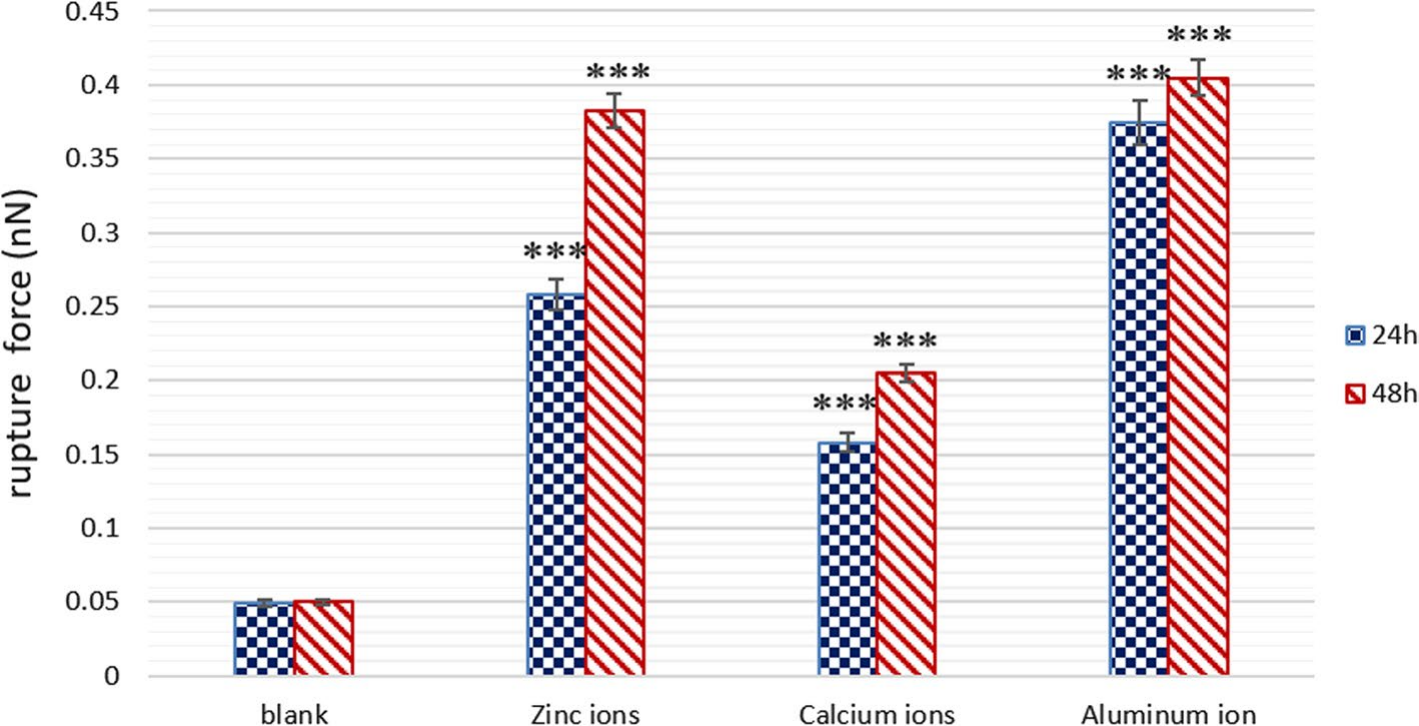
Bar plot summarizing the adhesion force between Aβ42 monolayers in the absence and presence of metallic ions at different incubation time points. All experiments were performed ten times (*n* = 10). ***P < 0.001 vs. control

Furthermore, the effect of metallic cations on Aβ42 interaction was investigated by measuring the rupture force in different conditions. Representative force–distance curves recorded in different groups are shown in Fig. 3. The force measurements were repeated 10 times for each group. Subsequently, the average rupture force for each sample is analyzed and shown in Fig. 6, which provides a clear comparison between the control group and experimental groups. The result of force test showed that in blank solution, the rupture force between Aβ42 monolayers was 0.049nN at the incubation time of 24 h and 0.05nN at the incubation time of 48 h, respectively. Whereas in the presence of Zn^2+^ the rupture force between Aβ42 monolayers was observed to increase to 0.258nN at the incubation time of 24 h and 0.383nN at the incubation time of 48 h, in the presence of Ca^2+^ the rupture force between Aβ42 monolayers was observed to increase to 0.158nN at the incubation time of 24 h and 0.205nN at the incubation time of 48 h. Moreover, in the presence of Al^3+^, significantly increased rupture force was recorded (0.375nN at the incubation time of 24 h and 0.405nN at the incubation time of 48 h). In conclusion, the results of force test showed that the interaction (adhesion) between Aβ42 monolayers increased with the addition of metallic ions in solution.

### Results of SERS

In general, the enhancement of the surface signal is 106 times, which is equivalent to the amplification of the surface monolayer to more than one million layers. Therefore, the advantage of SERS is that it can avoid the signal interference caused by the same substance in the solution, and obtain high-quality surface molecular vibration and rotation signals, which is of great significance for a detailed understanding of the interaction mode between molecules (such as metal ions) and self-assembled monolayers and the structural changes of molecules. Since the discovery of SERS, it has been successfully applied in many fields, such as chemistry and biology.

**Fig. 7:**
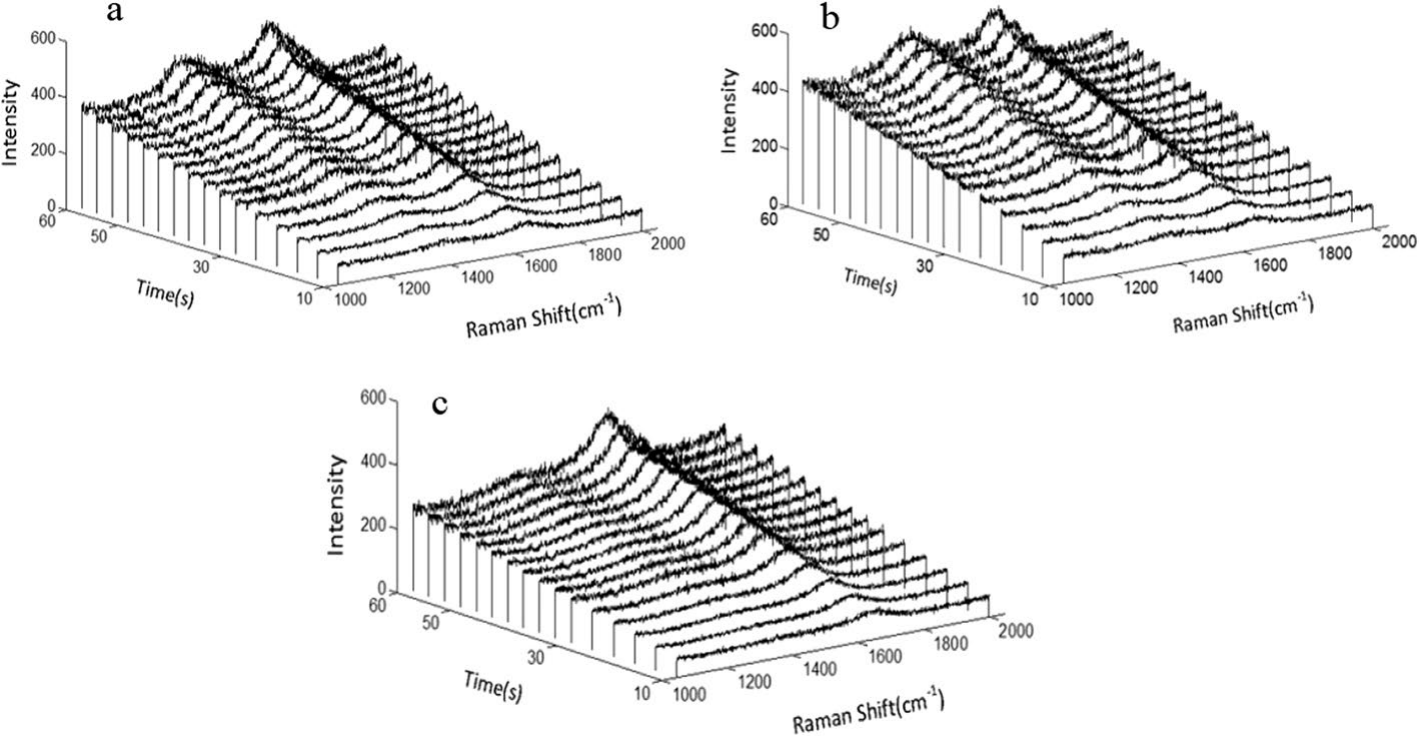
Raman spectrum curves for Aβ42 monolayer in the presence of Zn^2+^(**a**), Ca^2+^(**b**), Al^3+^(**c**)

Above all, stable SERS signal is of great significance for accurate analysis results. Hence, the SERS properties of the obtained Aβ42-modified substrates were evaluated in different metal ion environments. In order to investigate the stability of Raman measurement results, the changes of SERS spectra of Aβ42 molecular layer were recorded under continuous laser irradiation. As shown in Fig. 7, when different integral time was set, no obvious change in the peak shape of Raman spectrum curves for different samples was observed, which means the Aβ42 monolayer in these three metal ion solutions has good stability under continuous laser irradiation.

**Fig. 8:**
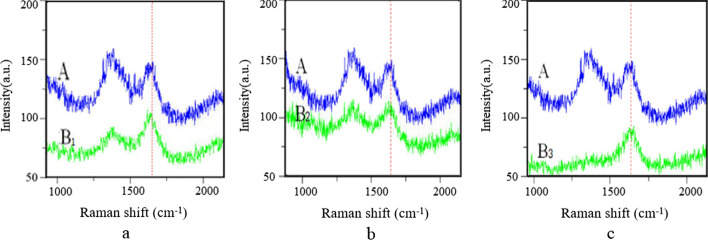
Raman Spectra about various metallic ions effects on Aβ42 (integration time for 10 s), with line A as control group; **a** in the presence of Zn^2+^, **b** in the presence of Ca^2+^, **c** in the presence of Al^3+^

**Fig. 9:**
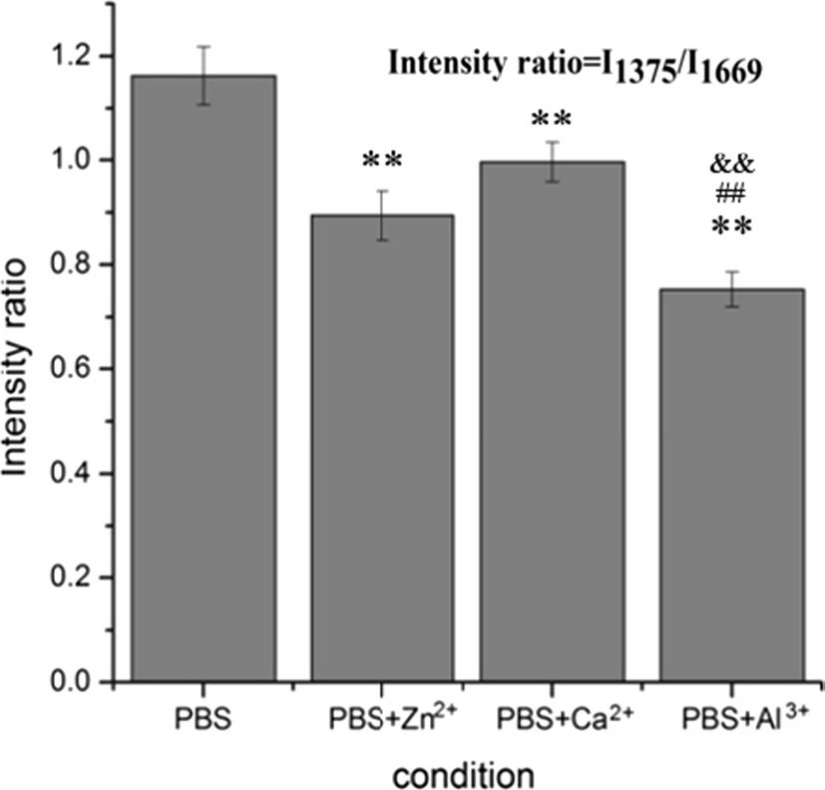
Intensity ratio I_1375_/I_1669_ of Aβ42 molecule in the absence and presence of metal cations (integration time for 10 s). All experiments were performed in triplicates (*n* = 3). **P < 0.01 vs. control (PBS); ^##^*P* < 0.01 vs. PBS + Zn^2+^; ^&&^*P* < 0.01 vs. PBS + Ca^2+^

In Fig. 8, curve A stands for the Raman spectrum of the blank control group and curve B stands for the experimental group. The results indicated that β-fold (peak at 1669 cm-1) and Amide II (peak at 1375 cm-1) are the characteristic structure for the natural conformation of Aβ42. With the addition of Zn^2+^, the peak intensity of the β-folded conformation at 1669 cm-1 and amide II band at 1375 cm-1 was weakened to a certain extent, respectively (Fig. 8(a)). Moreover, in blank control group, the Raman peak signal at 1375 cm-1 was determined to be stronger than that at 1669 cm-1, which means the vibration attributed to N–H is greater than that attributed to β-fold. On the contrary, the signal intensity of Amide II (1375 cm-1) was greatly weakened after adding Zn^2+^, which is lower than that of β-fold (1669 cm-1). As shown in Fig. 8(b), the addition of Ca^2 +^ also caused a structural change of Aβ42, which is characterized by the reduced Raman intensity of Amide II from 156.13 to 115.36, and the reduced Raman intensity of β-fold decreased from 144.669 to 117.76. It is worth noting that the Raman peak of Amide II at 1375 cm^−1^ was greatly weakened and almost disappear, and the intensity of the β-fold peak at 1669 cm^−1^ was also decreased compared with the blank control group, indicating that a considerable part of amide bonds in Aβ42 molecule probably interacted with Al^3+^ which resulted in a conformational change (Fig. 8(c)). Furthermore, the change of molecular conformation was evaluated by the Raman intensity ratio (*I*_1375_/*I*_1669_) (as shown in Fig. 9).

## Discussion

### Preparation of chemically immobilized Aβ42 monolayers

It is of crucial importance to consider immobilization carefully for the sensitivity and reproducibility of bioassays. Self-assembly monolayer (SAM), which is reliable for protein immobilization, is a topic of current interest in biological studies. SAM method can be used for self-assembly of protein molecules without altering the stability and activity of protein [26]. It has been studied that thiol concentration of 1 mM and immersion time of 24 h are befitting for the formation of Mercaptan molecular film [27]. Besides, MHA with a proper length of the chain serves as a spacer to minimize the interference from gold substrate [28].

We monitored the self-assembled processes by X-ray photoelectron spectroscopy (XPS) to ensure that Aβ42 was successfully modified on the surface of the gold substrate. Electron emission can be observed when the sample is exposed to electromagnetic waves with short enough wavelengths, i.e., high photon energy. This phenomenon is called photoelectric effect or photoionization because of the presence of observable photocurrents. In this process, the binding energy of material can be expressed by the following equation:

$$ E_{k} = hn - \, E_{b} - f_{s} , $$

*E*_*k*_ represents the kinetic energy of photoelectrons (in eV); *hν *represents the energy of photons in the X-ray source (in eV); *E*_b_ represents the binding energy in the specific orbit of an atom (in eV); *ϕ*_s_ represents the work function of the spectrometer (in eV).

Figure 2 shows that the elements on the surface of the three samples are identical to the modified molecules during self-assembly. XPS results demonstrated that for the MHA film and the Aβ42 monolayer, no noticeable chemical elements in addition to the ones expected based on their chemical constitution were found on the sample. The peak values of Au4f binding in the XPS spectra of blank substrate are 86.7 eV and 83.9 eV, respectively, which are consistent with the standard spectra. The peak values of Au4f binding shifted to 87.9 and 90.8 eV after the MHA molecule was modified on the surface of gold film. The formation of Au–S bond should be the cause of the binding energy shift of Au4f. What is more, peak position of the S2p binding energy spectrum (Fig. 2d) of the MHA film is lower than 164 eV, indicating that there is no unbounded MHA molecule on the sample surface [29]. There are two main peaks in the binding energy spectrum of S2p. The peak at 161.2 eV may be due to the bond between MHA and Au, which reduces the binding energy of S2p [30]. The peak at 161 eV may be attributed to the C-S bond [31]. The results suggest that the immobilization of Aβ42 on the gold substrate is successful.

### The aggregation behavior of Aβ42 observed by AFM

**Fig. 10:**
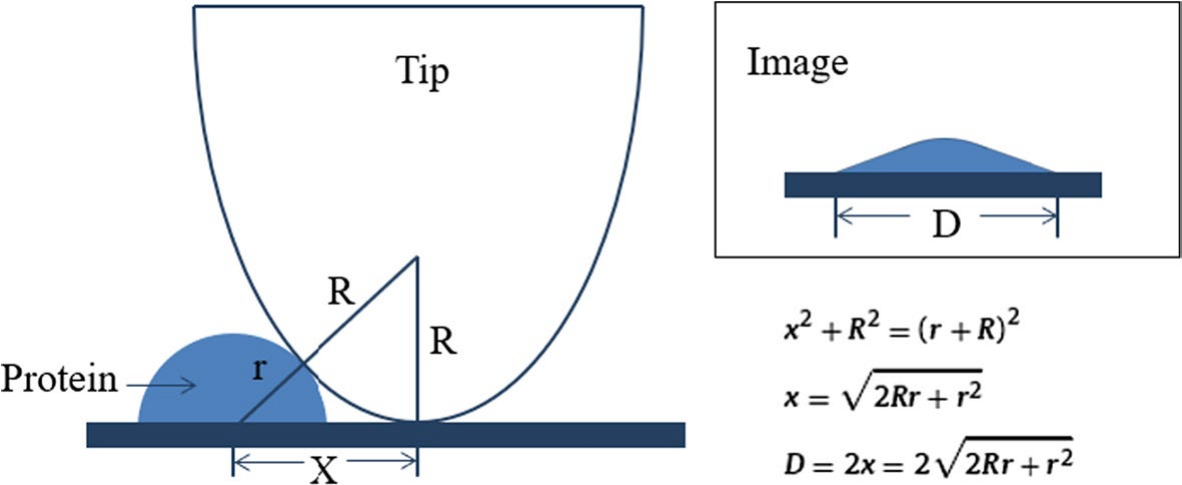
The broaden effect brought by AFM tip. R represents the tip radii, r represents the real width of protein structure, x and D represent the apparent widths of protein structure, respectively. The final recorded image with a broadened size is shown at the upper-right of the figure. The calculation formula is shown in the lower right corner

As for topography images recorded by AFM contact mode, it is worth noting that broaden effect introduced by the probe tip will enlarge the particle size (as illustrated in Fig. 10). Based on electron micrographs, a round shape for the probe tip is assumed. A half sphere structure is assumed as an Aβ42 molecule immobilized on the substrate. When a tip scans over the sample surface, it constantly approaches an elevation from the side of Aβ42 (or its aggregates) contour. The elevation with its outer edge will be recorded as the morphology of Aβ42 (or its aggregates) before the center of a probe tip reaches the real point where the elevation starts [32].

The average diameter of the nanostructures (D = 2x) on the topography images of the Aβ42 monolayer in the absence and presence of metallic ions was analyzed, respectively (as shown in Fig. 5). The average size of the observed nanostructure on the surface of Aβ42 monolayer in blank solution (PBS) at the incubation time of 24 h was found to be 43.12 nm (Fig. 5, blue bar in blank group). At the incubation time of 48 h, little change in particle diameter was observed. Considering the broaden effect according to equation in Fig. 8 for a tip of R = 25 nm (data given by the tip manufacturer), this real size for nanostructures in blank group is calculated to be ~ 16 nm, which is close to the theoretical estimation for size of Aβ42 oligomers [33]. With addition of Zn^2+^, the average diameter of the observed nanostructures increased to 65.87 nm at the incubation time of 24 h and 76.13 nm at the incubation time of 48 h, respectively. Considering the tip broaden effect, real size of Aβ42 aggregates is calculated to be ~ 32 nm at the incubation time of 24 h and ~ 40 nm at the incubation time of 48 h, which is more than double of the size of aggregates observed in blank group, indicating that Zn^2+^ promoted the mutual aggregation between Aβ42 molecules on the substrate. Compared to blank group, the real size of nanostructures on Aβ42 monolayer in the presence of Ca^2+^ increased to ~ 25 nm at the incubation time of 24 h and ~ 32 nm at the incubation time of 48 h, suggesting that the observed nanostructures in Ca^2+^ group are Aβ42 aggregates with higher aggregation levels. In addition, the real size of nanostructures observed in Al^3+^ group is the largest (~ 40 nm at the incubation time of 24 h and ~ 52 nm at the incubation time of 48 h), which is more than three times of the size of nanostructures in blank group, indicating that Aβ42 aggregation behaviors were promoted by Al^3+^. This observed size enlargement phenomenon suggest that more Aβ42 molecules react with neighboring Aβ42 molecules and formed Aβ42 aggregates due to the addition of metallic ions. In addition, combined with the mechanical test results, it can be further inferred that the observed aggregation behavior of Aβ42 molecules is a result of the increase in rupture force (adhesion) between Aβ42 molecules in the presence of metallic cations.

Moreover, it can be found that with the addition of metallic ions, the decrease of R_n_ occurred and R_n_ of Aβ42 monolayer in the presence of metallic ions also decreased in varying degrees with the increase of incubation time. We believe that the aggregation of Aβ42 particles leads to the collapse of molecular morphology, followed by more and more covered intermolecular gaps, eventually resulting in the decrease of R_n_.

Based on these findings, it can be suggested that in the presence of metallic ions, the aggregation behavior of Aβ42 was induced to varying degrees. In other words, the presence of metal ions leads to disorder and clumping on the surface of Aβ42 monolayer. What is more, stable aggregation state was observed during the experiment with the extending of incubation time. The results indicated that the addition of Zn^2+^, Ca^2+^, and Al^3+^ drastically destabilized Aβ42 and stabilized aggregation of Aβ42. The result is consistent with the mode proposed in Alies' paper [34]. Besides, the aggregation state of Aβ42 in 10 µM Zn^2+^, Ca^2+^, and Al^3+^solution differed in size of nanoparticles imaged by AFM. In conclusion, AFM imaging and force results showed large amounts of heterogeneous and conglobate-shaped aggregates were produced in the presence of metallic ions and the aggregation state of Aβ42 is stable due to the increased adhesion between Aβ42. The influence of different kinds of metal ions on the process of Aβ42 shows some differences, which may be attributed to the following reasons: (1) different charge amount, different molar ratio of Aβ42 to metal ions; (2) action modes and action sites on the Aβ42 peptide chain are different in the presence of different metal cations.

### SERS analysis

As shown in Fig. 7, the addition of different metallic ions leads to the decrease of the Raman strength ratio of I1 375/I 1669 to varying degrees. In comparison, I_1375_ / I_1669_ of Aβ42 monolayer in the presence of Al^3+^ is the lowest, which indicates that Al^3+^ make the greatest effect on the conformation change of Aβ42. The result is consistent with Banks’s study, suggesting that Al^3+^ can stabilize the aggregation structure of Aβ to a greater extent [35]. These results indicate that the main binding site of metallic ions in the process of Aβ42 aggregation should be the amide group of Aβ42 peptide chain (N–H bond) and the presence of metallic ions has a significant influence on the folding conformation of Aβ42 molecule.

The nanostructures with aggregation state on the surface in the presence of metallic ions is consistent with the results obtained by SERS. Based on these results, it can be inferred the presence of metallic ions plays a vital role in the occurrence of the abnormal aggregation events of Aβ42. The implications of this phenomenon are as follows: misfolding of Aβ conformation at the early stage leads to a destabilization and the interaction between metal cations and Aβ results in a conformational change of Aβ, which promotes formation of aggregates.

## Conclusion

A study of aggregation events between Aβ42 immobilized on MHA monolayer in the absence and presence of metallic ions by AFM imaging and SERS analysis is presented in this paper. The topographic images of Aβ42 monolayer either in the absence of metallic ions or in the presence of metallic ions (Zn^2+^, Ca^2+^, and Al^3+^) show significantly different surface structures. The obtained three-dimensional surface topography of Aβ42 monolayer show more pronounced state of aggregation in physiological solutions with added metal ions, which is characterized by the enlargement of nanoparticle sizes on the surface. The imaging results suggest that aggregation events occurred between Aβ42 molecules with the addition of metal cations. Furthermore, the effect of metal cations on the conformational change of Aβ42 was studied by SERS, which further supported the results obtained by AFM imaging. It can be inferred that the presence of metallic ions induces structural changes in Aβ42 and increases the intermolecular forces (adhesion) of Aβ42 and it is the increase of adhesion force that leads to a stable structure of Aβ42 oligomers and continuous molecular aggregation. These results, therefore, suggest that the presence of metallic ions plays a crucial role in the accelerated aggregation behavior of Aβ42. It is rational to assume that to block the effects from metal cations or obstruct the interaction between Aβ and metal cations may have great potential in new drug design for AD.

## Methods

### Materials

Metal salts used in this work are chlorides (Zncl _2_, Cacl _2,_ and Alcl_3_·6H_2_O), manufactured by Sigma Aldrich Chemical Co. All chemicals were used as received. Phosphate-buffered saline (PBS, pH 7.4) and absolute ethyl alcohol (guaranteed grade) were produced by Merck Co. Ultrapure water was made by Millipore purification system. 1-Ethyl-3-(dimethylaminopropyl)carbodiimide hydrochloride (EDC), N-hydroxysulfosuccinimide (NHS), and 16-mercaptohexadecanoic acid (MHA) are made by Sigma Aldrich Chemical Co. The amyloid peptides used in this work (Aβ42) were obtained from AnaSpec (USA).

### Formation of protein films

#### Preparation of gold film

In this experiment, vapor deposition method was applied to obtain the film of gold particles. First of all, a single layer of mica was gently peeled off in a sterile operating box by using a tweezer and the mica was then immediately placed in the radiant tube heater. Using a radiator heater, mica plates were heated to 325℃ for 2 h prior to deposition. After that, the preheated mica plate was moved into a turbo evaporator, where gold particle spray film deposited on the mica plate in high vacuum (at ~ 10^−7^ Torr). The velocity of evaporation is limited within the range of 0.1–0.3 nm/s. The thickness of gold granular film is about 200 nm. In addition, a chromium film was deposited between the gold and the surface of mica to increase the adherence. Finally, the obtained gold film was annealed in H_2_ flame for one minute. Finally, the Au-coated mica was divided into small squares of 1 × 1cm^2^.

Before use, the prepared Au-coated substrates were immersed in piranha solution (v/v H_2_SO_4_:H_2_O_2_ = 3:1) for 30 min to remove organic pollutants on the surface. The gold surface was then washed three times by absolute ethyl alcohol and ultrapure water in turn. At last, the surface of Au-coated substrate was dried in nitrogen to avoid any pollution. The surface of bare gold substrate was characterized by XPS.

#### Formation of MHA film

MHA was dissolved in an ethanol solution at a concentration of 1 mM. After that, the gold substrate was immersed into MHA solution to form MHA membrane spontaneously. Ultrasonic cleaning was applied to remove unbound MHA molecules. Subsequently, the SAM film was irrigated by absolute ethyl alcohol and ultrapure water successively. Finally, it is necessary to dry the MHA film in nitrogen immediately. The sample of MHA monolayer was characterized by XPS.

#### Aβ42 immobilization onto the MHA film

The carboxyl groups at the end of the MHA film reacts with the amino groups of lysine in Aβ42 molecule [36, 37]. Taking advantage of this principle, steady Aβ42 monolayer can be acquired [38]. The Aβ protein was dissolved in a physiological solution (PBS), which was freshly prepared according to standard method. It should be noted that MHA film were activated for 1 h at normal temperature using the method mentioned above. Afterward, the activated MHA film was rinsed as mentioned above and then immersed in 10 μM Aβ42 solution and stored in refrigerator at 4℃ for 12 h. At last, the Aβ42 film was prepared. Besides, the Aβ42-modified substrates should be rinsed three times by ultrapure water and dried in nitrogen before testing. This step is to remove free protein molecules from the surface of the substrate. The surface of Aβ42 monolayer was characterized by XPS.

### XPS

XPS experiments were conducted on a PHI Quantera SXM photoelectron spectrometer, which is equipped with an X-ray radiation source (Al Kα, 1486.6 eV). The photoelectrons were analyzed at a departure angle of 45°. Survey spectra were recorded in the range of 0–1400 e V. During the experiment, the pressure was controlled below 6.7 × 10^−8^ Pa. All XPS spectra were fitted by using an XPS peak-fitting program called Thermo Avantage.

AFM imaging and force measurement.

The powder of the three metal salts was dissolved in PBS, respectively, and diluted to 10 μM before use. For the comparison of Aβ42 aggregation events in the absence and presence of metal cations, the Aβ42 monolayers were incubated in blank solution (PBS) and metal ionic solutions (Zn^2+^ solution, Ca^2+^ solution, and Al^3+^ solution) and placed in 37℃ incubator for 24 h and 48 h, respectively. Three-dimensional images of the incubated Aβ42 monolayers (with scanning size of 1 μm × 1 μm) were achieved at a resolution of 512 × 512 and a scanning rate of 1 Hz. Surface topography of all samples were scanned by atomic force microscopy (JPK Nanowizard^@^II, Germany). Repeated cyclic scans were performed in CM-AFM. Three-dimensional topography of blank group and experimental groups recorded in different conditions was analyzed by imaging processing software which is provided by the manufacturer.

During force measurement, the functionalization of Au-coated probe tip with Aβ42 film was prepared similarly as described above. The Aβ42-modified tip scanned across the Aβ42 monolayer. At a random site on the sample, the tip approached the surface of Aβ42 monolayer and then retracted. 10 force–distance curves were collected for each sample. In this study, Commercial Si3N4 cantilevers with spring constant of 0.07 N/m (BudgetSensors, Innovative Solutions Bulgaria Ltd., Bulgaria) were used in contact mode of AFM (CM-AFM). The thickness of the gold layer on the tip is 70 nm.

### SERS determination

Aβ42 monolayer was prepared by the method mentioned above. 10 μM concentration of metal salts was dissolved in PBS solution. The prepared Aβ42 monolayer-modified substrate was placed in a clean liquid pool, 2 mL of blank solution (PBS), and metal ion solution (Zn^2+^, Ca^2+^, Al^3+^) was added, respectively, and immersed in an incubator (37 ℃) for 24 h.

The Raman spectra of freshly prepared Aβ42 monolayers were obtained from a confocal Raman spectrometer (LabRAM HR Evolution, HORIBA Jobin Yvon S.A.S. Co., France) with resolution of 1 cm^−1^. The laser was a He-Ne laser with excitation wavelength set at 633 nm, laser power set at 6.0mw, and the measurement range was 800 ~ 2800 cm^−1^. With PBS solution as blank control, the conformational changes of Aβ42 with the addition of metal cations were determined by Raman spectroscopy. For each experimental group, the Raman experiment was repeated at least three times.

In addition, we used Levenberg–Marquardt algorithm for peak split and fitting of the Raman spectral bands (corresponding to the β-folded conformation) (about 1600 ~ 1700 cm^−1^) and the characteristic peaks corresponding to amide bond at about 1400 cm^−1^. During the SERS testing, the same sample parameters were set. The integration time of Raman data were initially set as 10 s, and then the integration time was gradually increased to 60 s.

## Data Availability

The datasets used and analyzed during the current study are available from the corresponding author on reasonable request.

## Abbreviations

AFM: Atomic force microscope
SAM: Self-assembled monolayer
XPS: X-ray photoelectron spectroscopy
SERS: Surface-enhanced Raman Scattering
AD: Alzheimer's disease
Aβ: β-Amyloid peptides
MHA: 16-Mercaptohexadecanoic acid
EDC: 1-Ethyl-3-(dimethylaminopropyl)carbodiimide hydrochloride
NHS: *N*-Hydroxysulfosuccinimide
PBS: Phosphate-buffered saline
